# Evaluation of Enzyme Inhibitory Activity of Flavonoids by Polydopamine-Modified Hollow Fiber-Immobilized Xanthine Oxidase

**DOI:** 10.3390/molecules26133931

**Published:** 2021-06-28

**Authors:** Cong-Peng Zhao, Guo-Ying Chen, Yuan Wang, Hua Chen, Jia-Wen Yu, Feng-Qing Yang

**Affiliations:** 1 School of Chemistry and Chemical Engineering, Chongqing University, Chongqing 401331, China; 201818021136@cqu.edu.cn (C.-P.Z.); 201918021143@cqu.edu.cn (G.-Y.C.); 20136377@cqu.edu.cn (Y.W.); chenhuacqu@cqu.edu.cn (H.C.); 2 Taiji Group Chongqing Fuling Pharmaceutical Co., Ltd., Chongqing 408000, China

**Keywords:** xanthine oxidase, PDA-modified hollow fiber, enzyme immobilization, flavonoids, inhibitory activity

## Abstract

In this study, a polydopamine (PDA)-modified hollow fiber-immobilized xanthine oxidase (XOD) was prepared for screening potential XOD inhibitors from flavonoids. Several parameters for the preparation of PDA-modified hollow fiber-immobilized XOD, including the dopamine concentration, modification time, XOD concentration and immobilization time, were optimized. The results show that the optimal conditions for immobilized XOD activity were a dopamine concentration of 2.0 mg/mL in 10.0 mM Tris-HCl buffer (pH 8.5), a modification time of 3.0 h, an XOD concentration of 1000 μg/mL in 10.0 mM phosphate buffer (pH 7.5) and an immobilization time of 3.0 h. Subsequently, the enzymatic reaction conditions such as the pH value and temperature were investigated, and the enzyme kinetics and inhibition parameters were determined. The results indicate that the optimal pH value (7.5) and temperature (37 °C) of the PDA-modified hollow fiber-immobilized XOD were consistent with the free enzyme. Moreover, the PDA-modified hollow fiber-immobilized XOD could still maintain above 50% of its initial immobilized enzyme activity after seven consecutive cycles. The Michaelis–Menten constant (*K_m_*) and the half-maximal inhibitory concentration (IC_50_) of allopurinol on the immobilized XOD were determined as 0.25 mM and 23.2 μM, respectively. Furthermore, the PDA-modified hollow fiber-immobilized XOD was successfully applied to evaluate the inhibitory activity of eight flavonoids. Quercetin, apigenin, puerarin and epigallocatechin showed a good inhibition effect, and their percentages of inhibition were (79.86 ± 3.50)%, (80.98 ± 0.64)%, (61.15 ± 6.26)% and (54.92 ± 0.41)%, respectively. Finally, molecular docking analysis further verified that these four active compounds could bind to the amino acid residues in the XOD active site. In summary, the PDA-modified hollow fiber-immobilized XOD is an efficient method for the primary screening of XOD inhibitors from natural products.

## 1. Introduction

Xanthine oxidase (XOD) is a critical cytosolic oxidase, which is widely distributed in mammalian tissues [1]. It is a key enzyme of the purine pathway, which oxidizes hypoxanthine and xanthine to form uric acid while concomitantly producing hydrogen peroxide and superoxide anions [2]. Excess superoxide anions can interact with biomolecules such as lipids, proteins and DNA, thereby interfering in cell functions [3]. High concentrations of uric acid would result in hyperuricemia and gout [4]. Therefore, one of the most important therapeutic strategies is to effectively lower the uric acid level by inhibiting the XOD activity. Allopurinol is a potent inhibitor of XOD, which is used as a medication to treat hyperuricemia or gout in clinical practice [5]. However, many adverse reactions of allopurinol, such as allergic reactions, nephropathy and hepatitis, have been observed [6]. Hence, it is a significant issue to find new XOD inhibitors with high activity and low toxicity.

Flavonoids, which are extensively distributed in a variety of foods, are abundantly consumed in daily diets, such as in fruits, vegetables, tea and dietetic Chinese herbs [7]. Studies have demonstrated that flavonoids have various pharmacological activities, such as antioxidation, antiaging, anti-inflammatory, and antibacterial activities, as well as cardiovascular regulation [8]. In addition, flavonoids have been reported as potential XOD inhibitors and can effectively reduce uric acid in model animals [9,10,11]. Therefore, screening of XOD inhibitors from natural small-molecule compounds, such as flavonoids, can provide a reference for the development of drugs for the treatment of hyperuricemia and gout.

On the other hand, enzyme immobilization technology can immobilize water-soluble enzymes on the carriers that will not affect the enzymatic reactions [12]. It has advantages of enhanced enzyme stability, reusability, easy handling of enzymes, easy separation of enzymes from the reaction mixtures and prevention of enzyme contamination in the products [13,14,15,16]. According to the binding mode between enzymes and carriers, enzyme immobilization strategies can be classified into physical, chemical and affinity strategies. Physical strategies include adsorption and encapsulation, whose carriers do not covalently interact with the enzymes, while chemical strategies include covalent linking and cross-linking, in which there are covalent interactions between the carriers and enzymes. The affinity linkage strategy produces an affinity bonding between enzymes and a specific group of carriers [17]. Although physical adsorption and encapsulation are easy to be operated and have good enzyme activity, enzymes are easily leaked and have non-specific adsorption, while covalent linking and cross-linking have good reusability and high stability, but the enzyme activity is easily lost and cannot be recycled due to the tedious and time-consuming preparation process. The affinity immobilization strategy can achieve targeted immobilization and maintain good enzyme activity, but its immobilization requires some specific functional groups [12,17,18]. Therefore, one should choose the enzyme immobilization method in accordance with specific conditions.

In recent years, enzyme immobilization has frequently been used for enzymatic kinetics study and inhibitor screening [19]. Hollow fibers, which are a kind of organic polymer with a pore diameter and inner cavity, have unique characteristics including a large specific surface area, an abundant source, low consumption of biological material and organic solvents and very cheap equipment. Hollow fibers have been used in enzyme immobilization [20,21]. On the other hand, dopamine (DA, 3,4-dihydroxyphenethylamine) is a well-known molecular bio-mimetic adhesive with high biocompatibility and hydrophilicity, which can interact with various types of surfaces to form polydopamine (PDA) coatings due to its self-polymerization [22,23]. In addition, compared with conventional glutaraldehyde covalent immobilization, the enzyme activity could be enhanced using PDA modification [24].

The present study aimed to develop a rapid and efficient approach based on a PDA-modified hollow fiber-immobilized XOD (XOD@PDA@HF) to screen potential XOD inhibitors from flavonoids. The PDA-modified hollow fibers were prepared and characterized by Fourier transform infrared spectroscopy (FT-IR) and scanning electron microscopy (SEM). Subsequently, the enzymatic reaction conditions and the kinetics performance of XOD@PDA@HF were investigated. Finally, XOD@PDA@HF was used to evaluate the inhibitory activity of eight flavonoids, based on the mechanism that flavonoids interact with xanthine oxidase and inhibit the enzyme activity, and the amount of the generated product of the substrate hypoxanthine was used to evaluate the inhibitory activity on the enzymes of flavonoids. In addition, the binding sites and interactions between compounds and XOD were studied by molecular docking.

## 2. Results and Discussion

### 2.1. Characterizations

**Figure 1:**
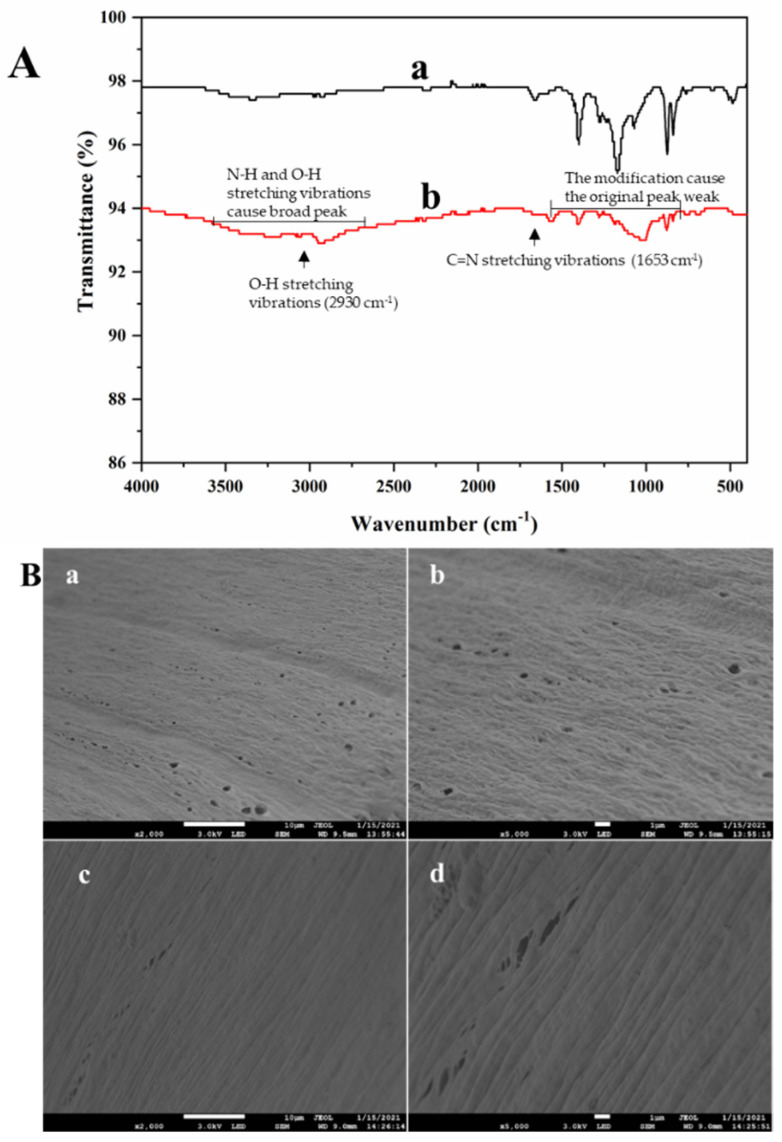
FT-IR spectra of hollow fiber and hollow fiber modified by PDA (**A**); (a) is hollow fiber and (b) is hollow fiber modified by PDA. SEM images of hollow fiber and hollow fiber modified by PDA (**B**); (a) and (b) are hollow fiber at 3.0 kV × 2000 and 3.0 kV × 5000, and (c) and (d) are hollow fiber modified by PDA at 3.0 kV × 2000 and 3.0 kV × 5000.

The FT-IR experiment was used to characterize the functional chemical groups on the hollow fiber, and the spectra are presented in Figure 1A. The PDA-modified hollow fiber exhibits O–H stretching vibrations at around 2930 cm^−1^ and C=N stretching vibrations at around 1631 cm^−1^. Additionally, the peak is broadened due to the stretching vibration of N-H and O-H at the range of 25,003,600 cm^−1^ compared to that of the pure hollow fiber. Some peaks are weakened at the range of 600–1600 cm^−1^, which can be explained by the PDA coating on the hollow fiber.

**Figure 2:**
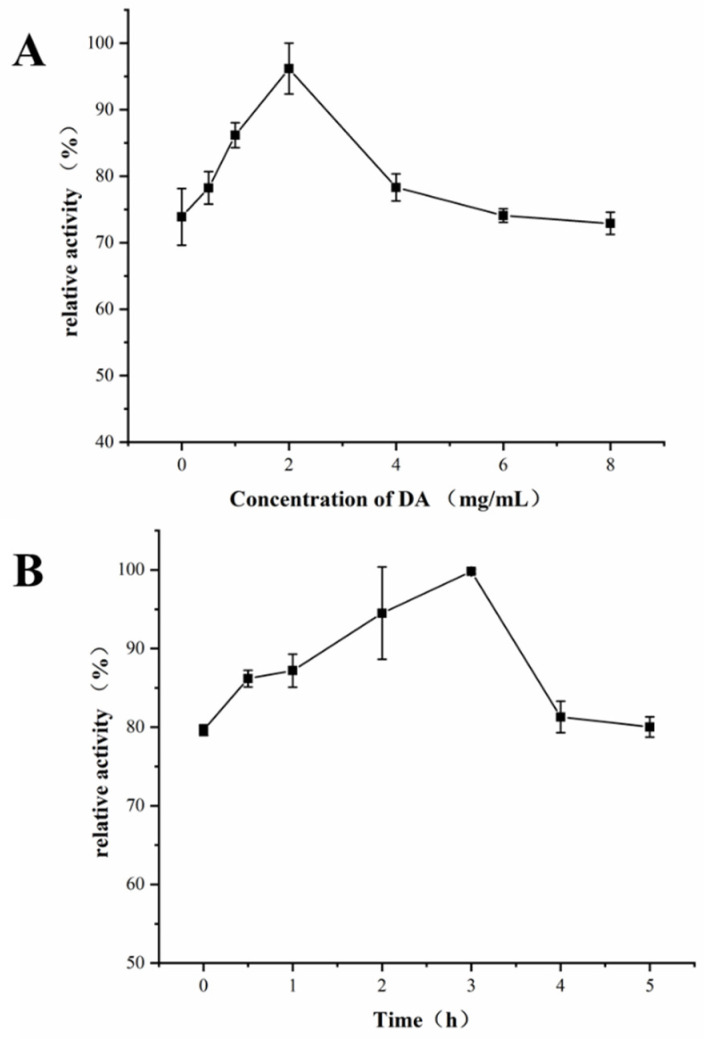
Effects of concentration of DA (**A**) and stirring time (**B**) on the activity of PDA@HF-immobilized XOD (the optimal activities of the immobilized enzyme counterpart were taken as 100%).

The pure hollow fiber and PDA-modified hollow fiber were further characterized by SEM. As shown in Figure 1B (a and b), the pure hollow fiber exhibited numerous large longitudinal stripes and cavities distributed on the fiber wall. Figure 2B (c and d) shows the images of the hollow fiber modified by PDA. After being modified by PDA, the large longitudinal stripes became tighter, and the cavities of the hollow fiber were reduced. In addition, the surface became smoother as compared to the pure hollow fiber. These results indicate that PDA formed a coating on the hollow fiber surface.

### 2.2. Optimization of Preparation Conditions of Hollow Fiber Modified by PDA

The hollow fiber was modified by PDA in Tris-HCl buffer solution (pH 8.5) of DA with magnetic stirring. To ensure the best activity of XOD@PDA@HF, the concentration of DA (0, 0.5, 1.0, 2.0, 4.0, 6.0 and 8.0 mg/mL) and the magnetic stirring time (0, 0.5, 1.0, 2.0, 3.0, 4.0 and 5.0 h) were optimized. As shown in Figure 2A, when the DA concentration was 2.0 mg/mL, the enzyme activity of PDA@HF-immobilized XOD was the highest. On the other hand, the stirring time is another important parameter that influences the hollow fiber modified by PDA. As shown in Figure 2B, the enzyme activity of PDA@HF-immobilized XOD reached the highest value at a stirring time of 3.0 h. Therefore, the optimal reaction conditions for the hollow fiber modified by PDA were a DA concentration of 2.0 mg/mL and a stirring time of 3.0 h.

### 2.3. Optimization of XOD Immobilization Conditions

**Figure 3:**
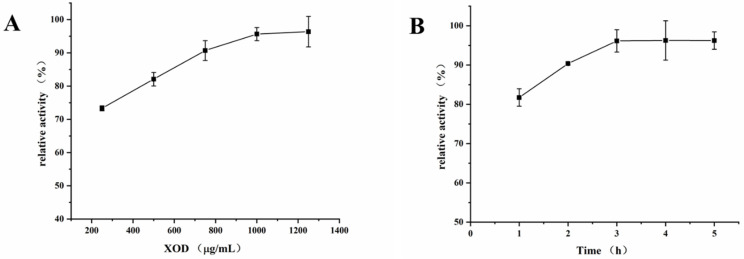
Effects of XOD immobilization time (**A**) and XOD amount (**B**) on the activity of PDA@HF-immobilized XOD (the optimal activities of the immobilized enzyme counterpart were taken as 100%).

The amount of XOD (250, 500, 750, 1000 and 1250 μg/mL) and reaction time (1.0, 2.0, 3.0, 4.0 and 5.0 h) in the preparation of XOD@PDA@HF were optimized. As shown in Figure 3A, the activity of XOD@PDA@HF increased when the XOD concentration was increased from 250 to 1000 μg/mL and reached the highest at 1000 μg/mL. Hence, 1000 μg/mL was chosen to be the XOD concentration for the immobilization. On the other hand, the reaction time is another important parameter that influences enzyme immobilization. As shown in Figure 3B, the activity of XOD@PDA@HF reached the highest value at 3.0 h of the reaction, which was used as the XOD immobilization time in the following experiments.

### 2.4. Effects of pH and Temperature on the Activities of Free and Immobilized XOD

**Figure 4:**
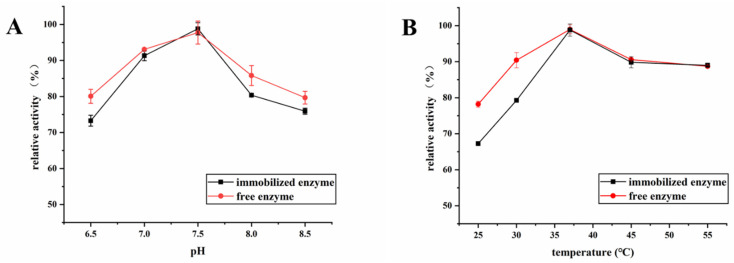
Effects of pH (**A**) and temperature (**B**) on the activity of free XOD and immobilized XOD (the optimal activities of the immobilized enzyme and free enzyme counterparts were taken as 100%).

The effect of the enzymatic reaction pH and temperature on the activities of immobilized and free XOD was investigated. The pH values from 6.5 to 8.5 were tested. As the results show in Figure 4A, the free enzyme’s activity was consistent with that of the immobilized one when the pH value was <8.5. The highest activities of free and immobilized XOD were both at pH 7.5, which indicates that the optimal reaction pH was not affected by the immobilization process. This may be due to the idea that PDA@HF-immobilized XOD does not change the protein conformation of enzymes. Furthermore, the effect of the temperature on the activities of free and immobilized XOD was investigated in the range of 25–55 °C. As shown in Figure 4B, the highest activity of immobilized XOD was achieved at 37 °C, which is also consistent with that of free XOD. Therefore, the PDA-modified hollow fiber has a relatively mild interaction force with xanthine oxidase that does not affect the optimal enzymatic reaction pH and temperature.

### 2.5. Performance of the XOD@PDA@HF

#### 2.5.1. Enzyme Kinetics Parameters

**Figure 5:**
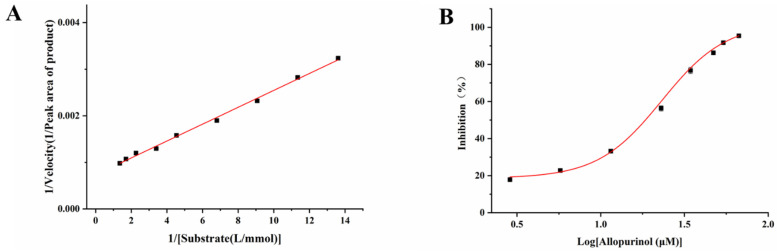
Double reciprocal plot at varied concentrations of substrate ranging from 0.07 to 0.7 µM (**A**). The inhibition curve for allopurinol on PDA@HF-immobilized XOD (**B**).

The Michaelis–Menten constant (*K_m_*), which was used to evaluate the affinity between the substrate and enzyme, was determined by measuring the peak area of the product at different substrate (hypoxanthine) concentrations. As shown in Figure 5A, the linear regression equation for XOD was Y = 0.00018X + 0.00073, with R^2^ = 0.9967, where X and Y are the reciprocals of the hypoxanthine concentration and reaction velocity, respectively. According to Equation (1), the *K_m_* value of the immobilized XOD was obtained from the slope and intercept, which was calculated to be 0.25 mM. This value is much lower than a previously reported *K_m_* value determined by an online capillary electrophoresis-based immobilized XOD microreactor (0.39 mM) using xanthine as the substrate [25], but it is higher than that of XOD immobilized on the internal walls of a fused silica capillary (14.5 µM) using xanthine as a substrate [26]. The reasons for the different *K_m_* values are the different substrates, the enzyme immobilization method and the enzymatic reaction conditions.

#### 2.5.2. Inhibition Kinetics Study

Allopurinol was used as a model compound to investigate the feasibility of XOD@PDA@HF in the inhibitory activity evaluation study. As shown in Figure 5B, the inhibition plot was established by varying the concentration of allopurinol at a fixed substrate concentration of 1.0 mM. The IC_50_ value could be obtained by constructing a dose–response nonlinear regression equation using Origin^®^8.5. The IC_50_ value (23.2 µM) determined in this report is slightly different from a previous study (27.2 µM) [27], which might be ascribed to the different strategy of immobilization, source of the enzyme and the enzymatic reaction conditions [28]. On the other hand, the *K_i_* value was calculated to be 4.6 µM through the Cheng–Prusoff equation, which is similar to that of a previous study (4.56 µM) [29].

#### 2.5.3. Reproducibility and Reusability

**Figure 6:**
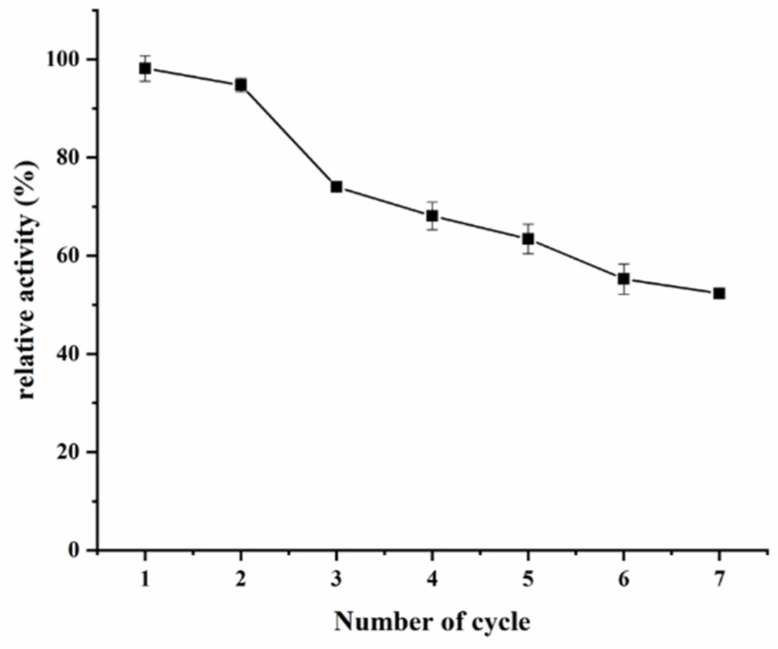
The reusability of XOD@PDA@HF.

The batch-to-batch reproducibility was investigated on three XOD@PDA@HFs prepared by the same procedure. The obtained relative standard deviation (RSD, *n* = 3) value of the peak area of products is 1.14%, indicating that it is a reliable and stable method for XOD immobilization. Furthermore, reusability of the immobilized XOD was demonstrated by its residual activities after seven cycles of usage. According to Figure 6, there was a decline in the residual activity of XOD@PDA@HF after repeated cycles, and it was retained above 50% of its initial immobilized XOD activity at the seventh cycle. This phenomenon could be explained by the enzyme shedding from the carriers as well as the denaturation of the enzyme.

### 2.6. Inhibitory Activity Evaluation of Flavonoids

The inhibitory activity of quercetin, apigenin, puerarin, catechin, epicatechin, epigallocatechin, epicatechin gallate and epigallocatechin gallate on XOD@PDA@HF was evaluated at a final concentration of 1.0 mM. Although 1.0 mM is a high concentration to test the inhibitory activity of compounds, it was used in this work to observe a strong inhibitory response on the immobilized enzyme. Further studies should be conducted in the future to test the effect of lower concentrations of inhibitors. The percentages of inhibition were calculated according to Equation (2), and the results are listed in Table 1. Quercetin, apigenin, puerarin and epigallocatechin have strong inhibitory activity on XOD, with percentages of inhibition of (79.86 ± 3.50)%, (80.98 ± 0.64)%, (61.15 ± 6.26)% and (54.92 ± 0.41)%, respectively. In reality, it was reported that some flavonoids such as quercetin, epicatechin and apigenin had inhibitory activity on XOD [25].

### 2.7. Molecular Docking Study

**Figure 7:**
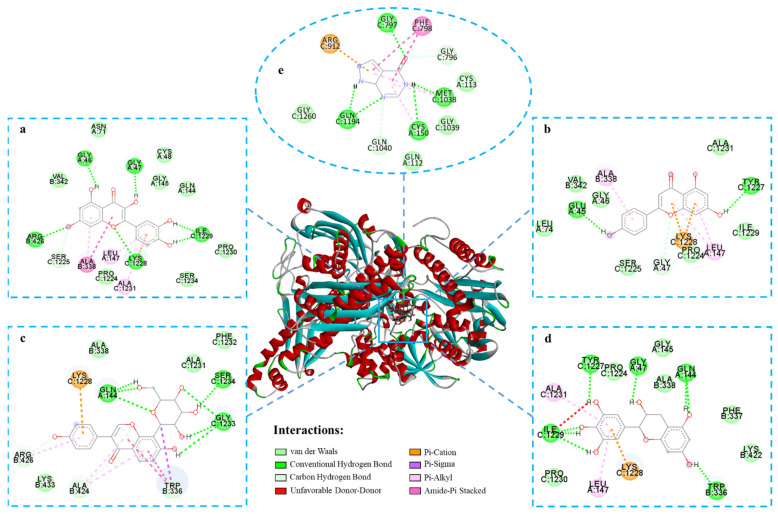
Molecular docking of quercetin (**a**), apigenin (**b**), puerarin (**c**), epigallocatechin (**d**) and allopurinol (**e**) with XOD.

The binding site and binding energy obtained by molecular docking analysis could be used for further verifying the results of the inhibitory evaluation assay. Four flavonoids (quercetin, apigenin, puerarin and epigallocatechin) with significant inhibitory effects and allopurinol were docked with XOD. Table 2 presents the binding energy and the hydrogen bonding of quercetin, apigenin, puerarin and epigallocatechin with XOD. Previous studies have shown that regions with binding energies below −5.0 Kcal could be considered to be potential targets [30]. The binding energy of allopurinol with XOD is −5.68 kcal/mol. Quercetin, apigenin, puerarin and epigallocatechin have similar binding positions to XOD, and their binding energies are all lower than −5.0 Kcal. Therefore, these four compounds may be potential XOD inhibitors. The docking results of these four compounds are shown in Figure 7. Quercetin, apigenin, puerarin and epigallocatechin act on the active center of XOD via multifarious interactions, including van der Waals forces, conventional hydrogen bonding, carbon hydrogen bonding and Pi–cation, Pi–sigma and Pi–alkyl forces. For example, quercetin could insert into the XOD binding pocket based on the interactions with residues of ARG426, LYS1228, ILE1229, GLY46 and GLY47 via hydrogen bonding, the interactions with residues of ASN71, CYS48, VAL342, GLY145, GLN144, PRO1230, PRO1224 and SER1234 via van der Waals contacts, the interactions with ALA338 via amide–Pi stacking and the interactions with LEU147 and ALA1231 via Pi–alkyl. These various interactions allow quercetin to bind to the active site of the enzyme, thereby achieving the good inhibitory effect on the enzyme activity.

## 3. Materials and Methods

### 3.1. Chemicals and Materials

The polyvinylidene fluoride fiber, MOF-1b, with an inner diameter of 0.5 mm and a pore size of 0.1 μm, was purchased from Tianjin Motianmo Engineering (Tianjin, China). XOD and hypoxanthine were purchased from Shanghai Yuanye Biotechnology Co., Ltd. (Shanghai, China). Allopurinol was purchased from Shanghai Macklin Biotechnology Co., Ltd. Tris(hydroxymethyl) aminomethane (Tris) was purchased from Sangon Biotech Co., Ltd. (Shanghai, China). Dopamine was purchased from Aladdin Chemistry Co., Ltd. (Shanghai, China). Epicatechin, epicatechin gallate, epigallocatechin, epigallocatechin gallate, quercetin, catechin, apigenin and puerarin were all purchased from Chengdu Biopurify Phytochemicals Ltd. (Chengdu, China). HPLC-grade methanol, acetonitrile and formic acid were obtained from Beijing InnoChem Science & Technology (Beijing, China). Water used for all the experiments was purified by a water purification system (ATSelem 1820A, Antesheng Environmental Protection Equipment, Chongqing, China). Unless specified otherwise, all the other chemicals and solvents, such as sodium chloride (NaCl) and hydrochloric acid (HCl), were all of analytical grade and purchased from Chengdu Chron Chemicals Co., Ltd. (Chengdu, China).

### 3.2. Instruments and HPLC-DAD Analysis

HPLC analysis was performed on an Agilent 1260 Series liquid chromatograph system (Agilent Technologies, Palo Alto, California, CA, USA), which was equipped with a vacuum degasser, a binary pump, an autosampler and a diode array detector (DAD), controlled by Agilent Chem Station software. An Agilent ZORBAX SB-Aq column (250 mm × 4.6 mm i.d., 5 μm) along with a pre-column (ZORBAX SB-Aq guard column, 12.5 mm × 4.6 mm i.d., 5 μm) was employed for the separation. An isocratic elution of solvent A (0.1% formic acid water solution) and solvent B (acetonitrile) was applied for the separation: 0–8 min, 10% B. The flow rate was set at 0.6 mL/min, detection wavelength was set at 290 nm, the column temperature was kept at 30 °C and the injection volume for all samples was 5.0 μL. An FE28 pH meter (Mettler-Toledo Instruments, Shanghai, China) was used for measuring the pH of solutions. The temperature control process was carried out using a drying oven (DHG-9146A, Longyue Instrument Equipment Co., Shanghai, China). A magnetic stirrer (HJ-4, Jintan Baita Xinbao Experimental Instrument Factory, Changzhou, China) was used for preparing the PDA@HF. In addition, a gas bath thermostatic oscillator (SHZ-82, Jintan Chengxi Zhengrong Experimental Instrument Factory, Changzhou, China) was used for preparing the XOD@PDA@HF.

### 3.3. Sample Preparation

Dopamine hydrochloride (2.0 mg/mL) was prepared in Tris-HCl buffer (10.0 mM, pH 8.5). The stock solution of XOD (1000 μg/mL) was prepared in phosphate buffer (10.0 mM, pH 7.5) and stored at −20°C. Hypoxanthine solution was prepared by dissolving it in phosphate buffer (10.0 mM, pH 7.5). Allopurinol was dissolved in phosphate buffer (10.0 mM, pH 7.5) to prepare a series of concentration (2.9–66.9 µM) solutions. The reference compound solutions of epicatechin, epicatechin gallate, epigallocatechin, epigallocatechin gallate, quercetin, catechin, apigenin and puerarin were prepared by dissolving them in phosphate buffer (10.0 mM, pH 6.8), with a final concentration of about 2.0 mM.

### 3.4. Preparation of XOD@PDA@HF

**Figure 8:**
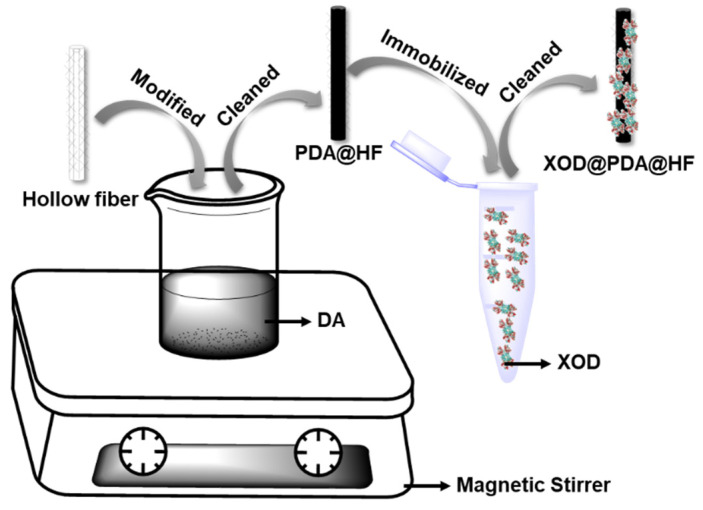
Schematic diagram of PDA-modified hollow fiber-immobilized XOD.

A schematic diagram of the XOD immobilization on the PDA-modified hollow fiber is shown in Figure 8. Firstly, the hollow fibers were pretreated. The hollow fibers were cut into a size of 2 cm and washed under ultrasonication successively by acetone, methanol and distilled water for 5 min. Secondly, the hollow fibers were modified by PDA. The hollow fibers were placed into to the DA solution and stirred by magnetic force for 3 h. PDA@HFs were cleaned three times by Tris-HCl before the next step of preparation. Finally, we prepared the XOD@PDA@HFs. For each experiment, fifteen cleaned PDA@HFs were placed in a 5 mL centrifuge tube containing XOD solution (50 U) and oscillated at 37 °C for 3.0 h with a 150 rpm oscillator. After immobilization, XOD@PDA@HFs were washed three times with phosphate buffer and stored at 4 °C in a refrigerator.

### 3.5. XOD Activity Assay

The enzymatic activities of free and immobilized XOD were assayed by the hydrolysis of hypoxanthine in phosphate buffer (10.0 mM). The concentration of the hydrolysis product (uric acid) was measured at 290 nm by HPLC. By taking the maximum activity of the free and immobilized XOD under optimal conditions as 100%, the activities in the other conditions were expressed as relative activities.

For evaluating the activity of free XOD, the enzymatic reaction was performed with a final volume of 0.5 mL, containing 10.0 mM phosphate buffer (pH 7.5), 0.05 mg/mL hypoxanthine and 11 U/mL XOD. The reaction solution was incubated at 37 °C for 5 min, and the enzymatic reaction was stopped by boiling water (99 °C) for 2 min. Subsequently, the reaction mixture was filtered through a 0.22 µm nylon membrane and injected into HPLC for analysis.

For evaluating the activity of XOD@PDA@HF, the reaction mixtures containing 0.5 mL 0.05 mg/mL hypoxanthine and an XOD@PDA@HF were incubated at 37 °C for 5 min. Finally, the XOD@PDA@HF was removed, and the solution was filtered through a 0.22 µm nylon membrane before HPLC analysis. The data were the average of at least three parallel experiments.

### 3.6. Characterization of the PDA@HF

The morphologies of the hollow fiber and PDA@HF were characterized by SEM (FESEM, JSM-7600F, JEOL Ltd., Tokyo, Japan). The chemical compositions of the prepared hollow fiber with and without modification by PDA were analyzed through a Nicolet iS50 Fourier transform infrared spectrometer (Thermo Fisher Scientific Inc., Waltham, MA, USA) and recorded in the range from 500 to 4000 cm^−1^.

### 3.7. Enzymatic Kinetic Study

The *K_m_* is an important characteristic kinetic constant used to evaluate the performance of immobilized enzymes and can be calculated by the Lineweaver–Burk equation, Equation (1) [31]:

(1) $$\frac{1}{V}=\frac{K_{m}}{V_{max}\left[S\right]}+\frac{1}{V_{max}}$$

where *V* and *V_max_* are the initial and maximum reaction velocities, respectively, while [*S*] is the concentration of substrate.

Under the optimal conditions, different concentrations of hypoxanthine (from 0.07 to 0.7 mM) were analyzed in triplicate, and the peak area of the product was used to represent the initial reaction velocities (*V*). The *K_m_* value of the enzymatic reaction was obtained by constructing the double reciprocal curve of 1/ [peak area of product] versus 1/[substrate] by Origin Pro 8.5 software.

### 3.8. Inhibition Kinetics Study of XOD

Allopurinol, a commercially available XOD inhibitor, was used as a model compound for evaluating the inhibition kinetics of the XOD@PDA@HF. IC_50_ was determined by varying the concentration of allopurinol (2.9–66.9 µM) at a constant substrate concentration of 1.0 mM. The inhibition percentage was calculated by following Equation (2) [32]:

(2) $$I\left(\%\right)=\left(1-\frac{A_{i}}{A_{0}}\right)\;\times \;100\%$$

where I(%) represents the percentage of inhibition, and A_i_ and A_0_ represent the peak area of the product obtained by enzymatic reaction with and without inhibitor, respectively. IC_50_ can be obtained from the dose–response nonlinear regression equation in Origin^®^8.5 and can be converted to inhibition constants (*K_i_*) according to the Cheng–Prusoff equation, Equation (3) [33]:

(3) $$K_{i}=\frac{IC_{50}}{1+\left(\left[S\right]/K_{m}\right)}$$

where *K_m_* is the Michaelis–Menten constant and [*S*] is the concentration of substrate.

### 3.9. Inhibitory Assay by XOD@PDA@HF

The constructed XOD@PDA@HF was applied in the inhibitory activity tests of eight flavonoids. Firstly, reaction mixtures containing 0.5 mL test solution and a XOD@PDA@HF were incubated for 10 min at 37 °C. Then, the reaction was initiated by adding 0.5 mL 0.05 mg/mL hypoxanthine and incubated for 5 min at 37 °C. Finally, the XOD@PDA@HF was removed, and the solution was filtered through a 0.22 µm nylon membrane before HPLC analysis. Blank controls consisted of phosphate buffer without testing solution. Furthermore, the percentage of inhibition was calculated according to the reduction in the peak area of the generated product uric acid compared with the peak area acquired without inhibitor (Equation (2)).

### 3.10. Molecular Docking Study

The AutoDock 4.2 program (The Scripps Research Institute, La Jolla, CA, USA) was employed for in silico molecular docking study to validate the binding potency of the compounds to XOD [34]. The docking operation was performed according to the following steps. Firstly, the crystal structure file of the XOD complex (PDB ID = 3ETR) was downloaded [35]. The dimension grid box (60 A × 60 A × 60 A) and grid spacing of 0.375 A were defined to enclose the active site. Secondly, the ligand was deleted using UCSF Chimera, unnecessary water molecules were removed and hydrogen atoms were added [36]. Thirdly, the 3D chemical structure of investigated compounds was drawn using Microsoft Office 3D and output in PDB format with minimized energy.

With the aim of docking with AutoDock Vina, the grid size was set to (x, y, z) = (60, 60, 60) and the grid center was set to (x, y, z) = (18.247, 0.33, 26.45). In each simulation process, progress with default parameters was run from AutoGrid and AutoDock, a Lamarckian genetic algorithm (LGA) was used to find the most favorable ligand binding orientations and the number of LGA runs was equal to 100. The interaction figures were generated, and the results of docking were recorded with binding energies and bonding residues.

## 4. Conclusions

In this study, XOD was efficiently immobilized on a PDA-modified hollow fiber for the first time. The PDA@HF-immobilized XOD has good activity and stability under an appropriate temperature and pH range. The PDA-modified hollow fiber-immobilized XOD could still maintain above 50% of the initial immobilized enzyme activity after seven successive cycles. Moreover, the PDA@HF-immobilized XOD was used to evaluate the inhibitory activity of eight flavonoids, and the results indicate that quercetin, apigenin, puerarin and epigallocatechin had strong inhibitory activity. Finally, molecular docking results further verified the binding energy and binding sites of the flavonoids and XOD. Although the traditional free enzymatic-based screen method has been proven to be an effective approach for discovering active compounds, it has the disadvantages of being time-consuming, laborious and inefficient. The developed XOD@PDA@HF is a simple, efficient and economical method for simultaneous screening of multiple targets from natural products. In other words, the developed XOD@PDA@HF is a potential tool for the primary screening of XOD inhibitors from natural products, which can provide a reference for the development of drugs for the treatment of hyperuricemia and gout. In addition, the PDA-modified hollow fiber can also be used to immobilize other enzymes for screening targeted inhibitors from natural products.

## Figures and Tables

**Table 1:** The percentage of inhibition of eight flavonoid compounds on XOD@PDA@HF.

Compounds	% of Inhibition	Compounds	% of Inhibition
Quercetin	79.86 ± 3.50	Epicatechin	16.61 ± 3.03
Apigenin	80.98 ± 0.64	Epigallocatechin	54.92 ± 0.41
Puerarin	61.15 ± 6.26	Epicatechin gallate	26.80 ± 5.78
Catechin	28.68 ± 0.60	Epigallocatechin gallate	33.07 ± 3.39

**Table 2:** Docking results of four flavonoids and allopurinol with XOD.

Compounds	Binding Energy (Kcal/mol)	Amino Acid Residues	Hydrogen Bonds
Allopurinol	−5.68	GLY796, MET1038, GLY797, GLN1040, CYS150, PHE798, ARG912, GLN1194	GLY797, GLN1194, CYS150, MET1038
Quercetin	−5.81	ALA338, GLY339, GLY46, GLY47, ARG426, SER1225, LYS1228, ALA1231, ILE1229, LEU147	GLY46, GLY47, ARG426, LYS1228, ILE1229
Apigenin	−6.74	GLU45, GLY47, LEU147, ALA338, TYR1227, LYS1228	GLU45, TYR1227
Puerarin	−6.54	GLN144, TRP336, GLY1233, SER1234, ARG426, ALA424, LYS1228	GLN144, SER1234, GLY1233
Epigallocatechin	−6.98	GLN144, TRP336, ALA1231, LEU147, GLY47, ILE1229, LYS1228, TYR1227	TYR1227, GLY47, GLN144, ILE1229, TRP336

## Data Availability

The data presented in this study are contained within the article.

## Sample Availability

Samples of the compounds are available from the authors.
